# Therapeutically Motivated Cannabis Use for Anxiety: Daily and Longitudinal Reductions Vary Between Flower and Edible Products

**DOI:** 10.3390/ijerph23020224

**Published:** 2026-02-10

**Authors:** Luiza Rosa, Jonathon K. Lisano, Carillon J. Skrzynski, Angela D. Bryan, L. Cinnamon Bidwell

**Affiliations:** 1 Department of Psychology and Neuroscience, University of Colorado Boulder, Boulder, CO 80309, USA; 2 Institute of Cognitive Science, University of Colorado Boulder, Boulder, CO 80309, USA

**Keywords:** cannabis, anxiety, flower, edible, cannabinoid, daily data

## Abstract

**Highlights:**

**Public health relevance—How does this work relate to a public health issue?**

- Many people use cannabis to cope with anxiety, but real-world daily effects are unclear.
- This paper tracked 30-day daily anxiety alongside product use, comparing flower vs. edible cannabis and different cannabinoid contents (THC-dominant, CBD-dominant, and THC + CBD).

**Public health significance—Why is this work of significance to public health?**

- Anxiety outcomes differed by cannabinoid content and method of use (flower vs. edible cannabis).
- CBD, especially in edibles, showed the most consistent anxiety reductions over time when participants used their products.

**Public health implications—What are the key implications or messages for practitioners, policy makers and/or researchers in public health?**

- Policy, research, and clinical guidance should distinguish THC vs. CBD and flower cannabis vs. edibles in messaging and regulation.

**Abstract:**

Research shows that delta-9-tetrahydrocannabinol (THC) is linked to increased anxiety, while cannabidiol (CBD) may have anxiolytic effects. Cannabis use is often driven by coping with anxiety, though its daily impact on anxiety remains unclear. This study examined daily associations between cannabis use and anxiety across 30 days in adults who wanted to use cannabis for anxiety relief. Participants (*N* = 345) used flower or edible products ad libitum and were randomly assigned to groups by product type (CBD, THC, or THC + CBD). Each day, participants reported cannabis use in the past 24 h and rated their anxiety. Linear mixed-effects models tested whether anxiety changed over time, differed by cannabinoid group, and varied with use. Anxiety significantly decreased over the study period in both flower and edibles groups. In the flower group, THC + CBD and CBD products had greater decreases in anxiety (39.5% and 34.8%, respectively) compared to THC products (7.8%). In the edibles group, when participants used CBD products, this was associated with a 24.9% reduction in anxiety over the 30 days. Findings underscore the importance of distinguishing cannabis effects by product type and cannabinoid composition and suggest that CBD-dominant edibles were associated with less anxiety over time in this naturalistic study.

## 1. Introduction

Anxiety-related reasons for cannabis use have become exceedingly common in recent years. Survey data indicate that anxiety, stress, and other mental health symptoms are among the most frequently cited motivations for both medical and non-medical cannabis consumption. For example, a 2017 nationally representative U.S. survey found that 49% of those using cannabis for medical purposes reported doing so for anxiety, making it the single most common medical motive for use, followed by insomnia (47%) and chronic pain (42%) [1]. Similarly, a broad meta-analysis of patient-reported motives for the use of medical cannabis concluded that roughly 50% of people who use medical cannabis cite anxiety as a key reason for use [2]. Outside of the medical sphere, a recent study of young adults noted that 73% of respondents using cannabis for health-related reasons cited anxiety, stress, and depression as reasons for cannabis use [3]. Together, this evidence indicates that managing feelings of anxiety is a prevalent motive for the use of cannabis.

The current literature regarding the impact of cannabis on anxiety is mixed. This may be due to the heterogeneity of cannabinoid ratios within cannabis products. The two most abundantly studied and well-known cannabinoids are Δ^9^-tetrahydrocannabinol (THC) and cannabidiol (CBD). In the context of anxiety, evidence points to opposing effects of THC and CBD. THC is the primary intoxicating component of cannabis and is known to be anxiogenic and produce related symptoms (e.g., panic or paranoia) at higher doses or in people who were inexperienced with cannabis use [4]. Childs and colleagues led a placebo-controlled study in people with moderate cannabis in which the dose-dependent effects of THC on anxiety were observed [5]. A 7.5 mg oral THC dose reduced subjective stress during a public-speaking task, while a slightly higher 12.5 mg dose actually increased anxiety and was associated with a more negative mood throughout the test. Consistent with this, recent work by our lab found that in a naturalistic, at-home use setting where participants self-selected their desired dose, acute cannabis use containing THC was associated with significant elevations in paranoia [6]. This underscores the nuanced and context-dependent effects of cannabinoids on anxiety-related outcomes. These findings highlight the varied effects in the literature, with some data pointing to THC being associated with higher levels of anxiety, while other data point to no harm or even potentially beneficial effects at lower doses.

Unlike THC, CBD is non-intoxicating and has garnered significant interest for its potential anxiolytic properties. Several studies suggest that CBD consistently reduces anxiety. Sharpe et al. conducted a systematic review that showed CBD consistently reduced anxiety without any negative effect at higher doses [4]. Further, CBD was associated with significant reductions in anxiety in a clinical population diagnosed with social anxiety disorder, particularly among individuals exposed to anxiety-inducing situations, such as public speaking tests [7]. Extending these findings, Bidwell et al. demonstrated that, in naturalistic settings, CBD-based flower cannabis products were associated with acute reductions in tension as well as greater decreases in anxiety symptoms across four weeks [6], highlighting both immediate and sustained anxiolytic effects.

Within the complexity of the individual impact of THC or CBD on anxiety, the interaction between their concurrent use is not well understood. Early studies suggested that CBD might decrease the anxiogenic effects of THC [7,8]. However, not all data support CBD countering the anxiogenic properties of THC. For example, in a double-blind, within-subject randomized trial, increasing the CBD to THC ratio (from 0:1 to 3:1) did not attenuate self-reported feelings of anxiety [9]. Thus, pharmacological research suggests that CBD can reduce anxiety on its own and, when combined with THC, may counteract some of THC’s adverse effects.

The route of cannabis administration (e.g., inhaling flower vs. consuming edible products) can drastically shape the onset, duration, and user control of effects, which in turn may influence the association of cannabis use with anxiety. Flower cannabis products have a rapid onset of action, in which those who use may feel effects within 5–10 min of inhalation [10], with effects lasting a couple of hours. Edible cannabis products exhibit a delayed onset, taking from 15 min to 3 h to manifest effects [10]. Differences in onset and duration between inhaled and edible cannabis products have important implications for anxiety management. For example, Stith et al. showed that flower cannabis provided significant short-term reductions in self-reported anxiety following use, allowing people using to self-titrate and stop once relief was achieved [11]. In contrast, edibles may be less impactful for acute anxiety due to their slow onset and prolonged effects, which can result in delayed or unexpectedly strong intoxication. One study found that edible or oral cannabis was associated with more frequent negative effects (i.e., feeling lazy, drowsy, or out of control) [12]. The delayed onset of edibles also increases the risk of overconsumption, as people who use it may ingest additional doses before the initial effects emerge, leading to more visits to medical professionals [13]. Although direct clinical comparisons are limited, the existing pharmacological and observational literature suggests that route of administration may play a significant role in the potential anxiolytic or anxiogenic effects of cannabis.

Despite the growing interest in cannabis for anxiety, much of the existing research is constrained by methodological limitations. The majority of existing research depends on retrospective self-report designs, wherein people are asked to recall their past cannabis use and anxiety symptoms (over weeks or months). Such approaches are vulnerable to memory and reporting biases [14,15]. Cross-sectional surveys provide only a static snapshot, making it hard to look at temporal sequences or within-person fluctuations. Laboratory studies, while more controlled, typically examine acute effects of a single dose of cannabis in an artificial setting, which may not generalize to how people use cannabis in real life. This points to a clear need for more real-world data on cannabis and anxiety using a daily diary methodology that is less vulnerable to recall bias and has high external validity.

The current paper presents an analysis of a preregistered [16] secondary outcome extending the primary longitudinal findings of Bidwell and colleagues [6]. Bidwell et al. showed that accounting for frequency of use, CBD-dominant flower cannabis was associated with the greatest reductions in anxiety over four weeks, whereas THC-dominant cannabis was associated with more modest improvements [6]. The sample includes participants who have used cannabis before, have mild-or-greater anxiety, and planned to use cannabis to mitigate their anxiety symptoms. Using 30 days of daily survey data from participants who used either flower cannabis or edible products ad libitum, this naturalistic daily diary study examined daily associations between cannabis use and anxiety symptoms. A preliminary aim was to examine whether usage frequency (i.e., number of daily use episodes) differed by product type (flower or edible) and chemovar group (THC, CBD, THC + CBD). The primary aim was to test whether daily cannabis use versus non-use was associated with same-day changes in anxiety, and whether these associations varied across time and cannabinoid ratio within the products being used (THC, CBD, THC + CBD). To evaluate potential differences by method of administration, this model was estimated separately for participants using flower and those using edibles.

We hypothesized first that participants using flower would report more frequent daily use episodes than those using edible cannabis products. We also hypothesize that daily cannabis use would be associated with same-day lower anxiety and that CBD-containing products would show the most consistent anxiolytic effects. Thus, the present study assessed, in flower and edible products, 1. if the level of anxiety changed over the 30 days of ad libitum use, 2. if these effects were cannabinoid group-dependent (THC, CBD, or THC + CBD), and 3. if using the product in the last 24 h was associated with anxiety that same day.

## 2. Materials and Methods

### 2.1. Participants and Study Design

This study employed a naturalistic, longitudinal daily diary design to examine within-person associations between cannabis use and anxiety over a 30-day period. Participants were recruited between March 2017 and December 2022 from the Denver/Boulder area through a combination of social media advertisements, mailed flyers, and community outreach events. Following completion of a pre-screening survey, participants were eligible if they had a score of 5 or higher on the Generalized Anxiety Disorder-7 scale (GAD-7; mild-or-greater anxiety), had at least one previous lifetime use of cannabis, and reported a desire to use cannabis to manage anxiety. Cannabis use during the study was self-directed (ad libitum), and no placebo or non-use control condition was included. A comprehensive list of inclusion and exclusion criteria is available elsewhere [6]. The larger study was pre-registered at Clinicaltrials.gov [16]. All procedures were approved by the University of Colorado Boulder’s Institutional Review Board (IRB #16-0767) and adhered to the ethical principles outlined in the Helsinki Declaration.

### 2.2. Procedures

Participants who met the eligibility criteria attended a baseline in-person laboratory session, where they completed informed consent and a battery of self-report questionnaires. At the end of this session, participants self-selected whether they preferred to use cannabis flower or edibles for the study duration. They were then randomly assigned to one of three groups based on cannabinoid content: (1) THC-dominant (flower: 24% THC, <1% CBD; edible: 10 mg THC, 0 mg CBD), (2) CBD-dominant (flower: <1% THC, 24% CBD; edible: 0 mg THC, 10 mg CBD), or (3) balanced THC + CBD (12% THC, 12% CBD; edible: 10 mg THC, 10 mg CBD). Randomization was determined via a pre-generated list by the study statistician, and all study staff involved in participant interaction remained blinded to group assignment.

Product formulations were selected to reflect commonly available options in Colorado, and participants independently purchased their assigned product from a local partner dispensary. Participants were asked to exclusively use their assigned product throughout the 4-week study but could choose dose, timing, and frequency of use based on their usual consumption patterns. To verify product fidelity, participants uploaded photos of product labels via REDCap (version 15.5.32) [17], a secure data storage system, which were reviewed by blinded study staff.

All participants were provided with safety guidance, including materials from the Colorado Marijuana Enforcement Division. Given the potential for THC-related adverse effects such as anxiety exacerbation, paranoia, or physiological discomfort, participant safety was monitored throughout the study period by a licensed clinical psychologist. Participants were informed that they could withdraw and obtain referral to appropriate care in the event of clinically significant anxiety, distress, or other impairing effects. No participants were withdrawn for clinical or safety-related reasons.

Participants were compensated with $80 in cash at the baseline visit. Before leaving the baseline session, participants were oriented to the daily survey protocol and completed practice entries via their smartphones or a lab device. They then received daily survey links for 30 days via REDCap, where they reported on cannabis use, product characteristics, and anxiety symptoms. Participants received $1 per completed daily survey, and if participants completed at least 80% of the daily surveys, they received a compensation bonus of $10. Compliance was tracked, and participants who missed two consecutive surveys were contacted by study staff to support continued engagement.

### 2.3. Measures

#### 2.3.1. Baseline Visit

Demographic and baseline clinical information, including age, gender, education level, employment status, and race/ethnicity, were collected during this initial visit.

Generalized Anxiety Symptoms: Symptoms of generalized anxiety were assessed using the 7-item self-report questionnaire the Generalized Anxiety Disorder-7 (GAD-7) [18]. Participants rated how often they were bothered by each symptom over the past two weeks using a scale from 0 (“Not at all”) to 3 (“Nearly every day”). Total scores range from 0 to 21, with 0–4 typically indicating minimal anxiety, 5–9 mild anxiety, 10–14 moderate anxiety, and 15–21 severe anxiety.

Substance Use and Expectancies: Cannabis and alcohol use frequency over the past 30 days was measured using the Online Timeline Followback (O-TLFB), a calendar-based self-report method that captures the number of days each substance was used [19]. Cannabis Use Disorder (CUD) symptoms were evaluated using a modified version of the Marijuana Dependence Scale (MDS) [20,21]. This scale contains 11 items with options varying between yes (1) and no (0) to assess the presence of CUD symptoms experienced over the past 12 months. Total scores reflect the number of criteria met and range from 0 to 11, with higher scores indicating more problematic use. Participants’ expectations that cannabis would alleviate anxiety were assessed using a single item, asking “Which of the following benefits do you expect to get from cannabis? (Please select the level of change you expect).” Response options included “decreasing anxiety” and ranged from 0 (“No improvement at all”) to 3 (“Very improved”) and were reverse-coded so that higher scores reflected stronger expectancies for cannabis to relieve anxiety.

#### 2.3.2. Daily Surveys (30 Days)

The daily surveys were delivered via REDCap and assessed whether participants used their assigned cannabis product in the past 24 h, the number of use occasions during that period, the estimated dose consumed, and feelings of anxiety.

Cannabis Use: Participants were asked whether they chose to use their assigned cannabis product in the past 24 h (yes or no). If they answered yes, they were further asked about how many separate times they had used their product.

Current anxiety: Participants were asked “How much anxiety, on average, did you experience in the past 24 h?” They rated this on a scale of 0–10 with (0) representing no anxiety and (10) representing the worst imaginable anxiety.

### 2.4. Statistical Analysis

Analyses excluded participants who completed less than 70% of daily surveys throughout the 30-day period, similar to previous studies [22,23,24]. All statistical procedures and visualizations were conducted in R [25] using a range of packages, including dplyr, sjPlot, ltm, nlme, emmeans, and ggplot2 [26,27,28,29,30,31]. Group-level differences in baseline variables such as age and cannabis expectancy were evaluated using ANOVAs, while chi-square tests were used to examine group differences in gender, education, employment, and ethnicity. Additional ANOVAs were conducted to examine differences in total days of use and average uses per use day.

Given known differences in pharmacokinetics, onset, and duration of effects between smoked and ingested cannabis, a first model examined whether usage frequency (i.e., number of use episodes per day) differed by method of administration (flower vs. edible) and cannabinoid group. Because our primary analyses already included a complex three-way interaction among time (study day), cannabinoid group (THC, CBD, and THC + CBD), and daily use (use vs. non-use), the models were run separately by method of administration to facilitate interpretation of these effects. To examine associations between daily cannabis use and anxiety, identical linear mixed-effects models were estimated separately for participants who used flower and those who used edible products. Each model included the fixed effects of time (study day), cannabinoid group (THC, CBD, or THC + CBD), and daily use (use vs. non-use), as well as their two- and three-way interactions. All models included random intercepts for participants to account for within-person dependencies and were estimated using restricted maximum likelihood. An autoregressive correlation structure was used in these models to account for temporal autocorrelation in daily anxiety ratings, under the assumption that observations closer in time are more strongly correlated than those further apart [31,32,33,34]. When significant interactions emerged, follow-up trend analyses were performed using the emtrends function from the emmeans package. Relevant pairwise comparisons were carried out using the pairs function. All models accounted for subject-level variability through random intercepts to capture within-person correlations over time. Random slopes for time were included to allow for individual differences in change trajectories over the study period, reflecting the possibility that the relationship between time and outcomes may vary from person to person. Each linear mixed-effects model controlled for covariates of cannabis anxiety expectancies, age, gender, employment, and education. These covariates were included to account for confounding variables that may influence both cannabis use and anxiety symptoms, allowing the models to provide a more accurate estimation of the primary associations.

## 3. Results

### 3.1. Descriptive Information

**Figure 1:**
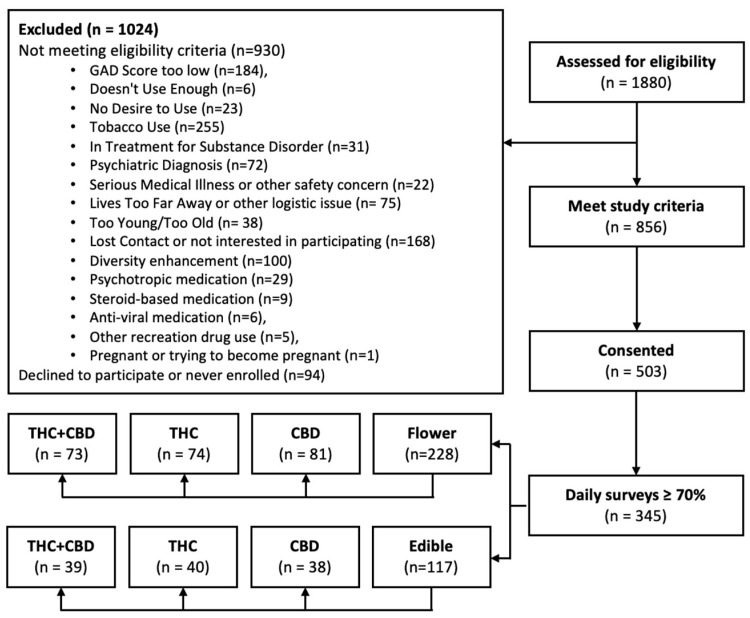
Participant CONSORT.

Of the 1880 participants who screened to participate in the study, 856 met the study inclusion criteria, 503 of whom consented to participating, and a total of 345 participants had at least a 70% completion rate for the daily study surveys (see Figure 1). Attrition analyses comparing baseline characteristics between retained and excluded participants indicated no significant differences in baseline cannabis use, anxiety, age, gender, education, or employment status (all *p*s > 0.32). Of the 345 participants, 228 selected flower and 117 selected edibles. Of these participants, 73 were assigned to the THC + CBD flower condition, 39 to the THC + CBD edibles condition, 74 to the THC flower condition, 40 to the THC edibles condition, 81 to the CBD flower condition, and 38 to the CBD edibles condition. Participants completed an average of approximately 27–28 of the 30 daily surveys (flower: *M* = 27.5, *SD* = 2.66; edible: *M* = 27.8, *SD* = 2.58), with a median completion of 28 days for those using flower and 29 days for those using edibles.

Across both methods of use, participants were predominantly White (74.6%) and identified primarily as female (61.3%) or male (36.0%), with a small proportion identifying as non-binary or preferring not to disclose gender. Employment status was diverse, with roughly 40–45% reporting full-time employment and an additional 20–25% working part-time; smaller proportions identified as full-time students, unemployed, or homemakers. At baseline, there were no significant differences by method of use in GAD-7 scores (*F* (2, 321) = 1.75, *p* = 0.18) and, on average, participants reported moderate anxiety (overall mean GAD-7 = 11.60 ± 4.19). There were no significant differences in cannabis anxiety expectancies at baseline by condition observed (*F* (2, 339) = 0.08, *p* = 0.925). However, we did see that the flower group was significantly younger (Flower: 30.62 ± 11.38; Edible: 36.95 ± 14.61; *F* (1, 336) = 19.29, *p* < *0*.001) and had more problematic cannabis use as measured by the Marijuana Dependence Scale (MDS) than the edibles group (Flower: 2.07 ± 2.30; Edible: 0.77 ± 1.42; *F* (1, 331) = 29.75, *p* < *0*.001). The edible cannabis group also had more females and was more educated than the flower group. No differences were observed regarding method of use. Detailed participant demographics and characteristics can be found in Table 1, Table 2 and Table 3.

### 3.2. Method of Administration and Use Frequency

A preliminary model investigating whether the route of administration (flower vs. edible) and cannabinoid (THC-dominant, CBD-dominant, or THC + CBD) predicted use intensity, operationalized as the number of cannabis use occasions per day on days when cannabis was used (excluding non-use days). The results show a significant main effect of product type, with participants using flower reporting a greater number of use occasions per use day (*M* = 1.79, *SE* = 0.06) than those using edibles (*M* = 1.23, *SE* = 0.08), *p* < 0.0001. No other effects were significant (*p*s > 0.36). Descriptive statistics for total cannabis use occasions by method and cannabinoid composition over the study period are presented in Table 3. Given these differences in use intensity across methods of administration, subsequent analysis were run separately for flower and edibles groups.

### 3.3. Cannabis Flower and Daily Anxiety

**Figure 2:**
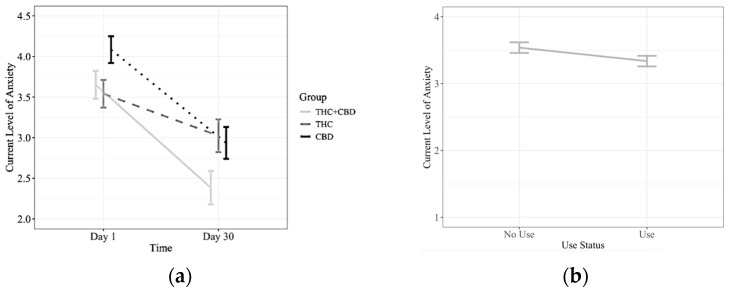
Flower Cannabinoid Group, Time and Use Graphs. Note: On the left, (**a**) shows flower cannabinoid group × time interaction *F* (2, 6027) = 2.93, *p* = 0.053. CBD (*β* = 0.03, *95% CI* [−0.03, −0.02], *p* = 0.04) and THC + CBD (*β* = −0.04, *95% CI* [−0.05, −0.004], *p* = 0.02) exhibited steeper reductions in anxiety compared to the THC group. No differences were observed comparing CBD and THC + CBD. On the right, (**b**) shows the use status main effect, showing no differences between the use and no-use conditions *β* = −0.23, *95% CI* [−0.55, 0.09], *F* (1, 5973) = 1.85, *p* = 0.17.

We examined whether anxiety levels differed on days when participants used their assigned flower product compared to days in which they did not use their product, and if these associations varied by cannabinoid group or across the study duration (Day 1 to Day 30). While there was no significant three-way interaction between use, group, and time (*p* > 0.05), there was a significant main effect of time (*β* = −0.02, 95% *CI* (confidence interval) [−0.03, −0.001], *F* (1, 6027) = 35.41, *p* < *0*.0001), indicating that anxiety decreased across the 30-day study period. The group × time interaction did not reach the traditional threshold for significance (*F* (2, 6027) = 2.93, *p* = 0.053). Follow-up analyses indicated that all three groups (THC-dominant, CBD-dominant, and THC + CBD) showed significant reductions in anxiety over time (Figure 2). However, when comparing the change over time in these groups, participants in the THC + CBD group experienced a greater rate of decrease in anxiety compared to the THC-dominant group (*β* = −0.04, 95% *CI* [−0.05, −0.004], *p* = 0.02). A similar pattern emerged with participants in the CBD-dominant group when compared to the THC-dominant group (*β* = 0.03, 95% *CI* [−0.03, −0.002], *p* = 0.04). There was no significant difference between the change in anxiety over time for the CBD and THC + CBD groups (*β* = 0.006, 95% *CI* [−0.02, 0.03], *p* = 0.89). To provide context for the magnitude of these time effects, the percent change in anxiety from Day 1 to Day 30 was calculated for participants. Among those using flower, the THC + CBD group mean anxiety decreased from 4.50 at Day 1 to 2.72 at Day 30, corresponding to a 39.5% reduction based on sample means. Similarly, in the CBD group, mean anxiety decreased from 4.03 to 2.62, corresponding to a 34.8% reduction. In contrast, the THC group showed smaller changes, with mean anxiety decreasing from 3.81 to 3.51 (7.8% reduction).

### 3.4. Cannabis Edibles and Daily Anxiety

**Figure 3:**
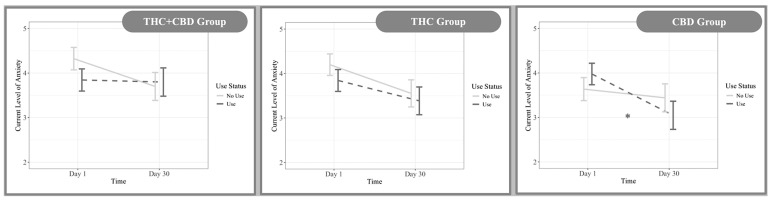
Edibles group × time × use interaction. Note: There was a significant three-way interaction between group × time × use (*F* (2, 3124) = 3.86, *p* = 0.02). Those who used their product in the CBD group reported significant reductions in anxiety from Day 1 to Day 30 (*β* = −0.05, *95% CI* [−0.08, −0.01], *p* = 0.009), indicated by “*” in the third panel. No other groups had significant interactions.

In the same manner as the model for flower, this model examined whether anxiety levels differed on days when participants used their assigned edible product compared to days in which they did not use their product, and if these associations varied by cannabinoid group or across the study duration (Day 1 to Day 30). Within this model, there was a significant main effect of cannabis use on anxiety (*β* = −0.50, 95% CI [−0.90, −0.09], *F* (1, 3124) = 5.89, *p* = *0*.02). This main effect showed that on days that participants used their edible cannabis product, anxiety was lower when compared to when participants did not use their product. A significant main effect of time also emerged, with anxiety declining through the 30-day study period, (*β* = −0.02, 95% CI [−0.04, −0.009], *F* (1, 3124) = 4.31, *p* = 0.04). Finally, there was also a significant three-way interaction between group, time, and use (Figure 3: *F* (2, 3124) = 3.86, *p* = 0.02). Among participants using edible cannabis, those in the CBD-dominant group reported significant reductions in anxiety from Day 1 to Day 30 (*β* = −0.05, 95% CI [−0.08, −0.01], *p* = 0.009). This association was not found in the THC-dominant or THC + CBD groups (*p* > *0*.05). To also contextualize the magnitude of anxiety change among those using edible cannabis products, the percentage change in anxiety from Day 1 to Day 30 was also examined. In the THC + CBD group, mean anxiety remained relatively stable from 4.27 at Day 1 to 4.30 at Day 30 (0.6% increase). In the THC group, mean anxiety decreased from 4.46 to 3.58, corresponding to a 19.9% reduction. In the CBD group, mean anxiety decreased from 4.31 to 3.24 (a 24.9% reduction).

## 4. Discussion

The present study examined whether cannabis use was associated with daily reductions in anxiety across a 30-day period, with a focus on cannabinoid (THC, CBD, THC + CBD) and method of administration (edibles vs. flower), in participants with mild or greater self-reported levels of anxiety who were interested in using cannabis for anxiety coping. We expected that daily cannabis use would be associated with decreased anxiety, that CBD-containing products would provide the most consistent anxiolytic effects. Overall, the results largely supported these hypotheses: in both product groups, cannabis use was significantly associated with within-person decreases in anxiety. To our knowledge, this is the first study to examine the day-to-day associations between cannabis use and anxiety among individuals who use legal market cannabis to cope with anxiety, providing novel insights into within-person variability and temporal dynamics of route of administration, cannabinoid ratios, frequency of use, and anxiety. Although prior daily diary studies of cannabis use have examined related outcomes such as sleep or general mood [35,36,37], these studies have not focused on anxiety as a primary outcome in a clinically relevant anxiety sample, nor have they directly looked at different routes of administration. In addition, previous work has often relied on medically prescribed products or been limited by sample size [36]. By capturing repeated observations in participants’ natural environments, these daily analyses enhance ecological validity and allow for a more precise understanding of how cannabis effects fluctuate across days and contexts.

### 4.1. Flower

Findings for cannabis flower showed robust overall reductions in daily anxiety across the 30-day study, with participants in CBD and THC + CBD groups experiencing steeper declines compared to the THC group (Figure 2). While all three flower groups demonstrated decreases over time, the THC-dominant group showed a more modest and less consistent pattern of anxiety reduction relative to the CBD-containing groups. Importantly, however, THC-dominant products were still associated with lower anxiety on use days, and we did not observe higher anxiety following same-day THC flower use. This pattern differs somewhat from prior laboratory work showing that THC can produce anxiety-related effects in controlled settings [4,5]. By contrast, the CBD and THC + CBD flower groups showed more sustained declines across the 30-day period, aligning with research suggesting that CBD may buffer or counteract the anxiogenic potential of THC while contributing its own anxiolytic properties [7]. These results also converge with Bidwell et al. who showed flower products containing CBD as most beneficial for anxiety [6]. The current paper advanced the work by incorporating within-person analyses to capture how cannabis use at specific cannabinoid ratios relates to day-to-day fluctuations in anxiety.

### 4.2. Edibles

For edible cannabis, results indicated a significant main effect of use on anxiety. Participants reported lower anxiety on days when they used their assigned edibles compared to non-use days. Additionally, anxiety declined over the 30-day study period, with a group × time × use interaction showing that the effects of cannabis use on anxiety changed over time and differed by cannabinoid group. Specifically, participants using CBD edibles showed the most consistent reductions in anxiety from Day 1 to Day 30, and the difference between use and non-use days became more pronounced over time in this group (Figure 3). These patterns may relate to the pharmacokinetics of edible cannabinoids. Unlike inhaled cannabis, edible THC and CBD have delayed onset, slower absorption, and greater variability in subjective effects [12,13]. This variability can lead individuals to modify the timing and pattern of their use as they gain experience with a product. It is plausible that participants in the edibles group became more adept at timing and calibrating their consumption across the 30-day period, resulting in increasingly reliable anxiolytic effects. This interpretation aligns with prior work showing CBD’s anxiolytic properties [4,7] and extends findings from Bidwell et al., adding support of the anxiolytic properties of CBD within edible products [6]. In contrast, THC and THC + CBD edibles groups showed less reliable anxiety reductions, consistent with evidence that oral THC can result in variable and sometimes anxiogenic effects due to delayed onset and less predictable pharmacokinetics [12,13].

### 4.3. Comparison Across Methods of Use

In looking at both models, results indicated that both flower and edibles were associated with within-person reductions in daily anxiety, but the effects of use differed. Participants using flower reported more frequent daily use occasions, potentially compensating for its shorter-lived effects [10], whereas those using edibles showed fewer use occasions throughout the study yet experienced more same-day anxiety reductions when they did use their study product. Importantly, only edibles demonstrated a significant interaction with use, such that using their product in the last 24 h, particularly of CBD edibles, was linked to steeper declines in anxiety over time. By contrast, flower-related reductions appeared more stable across the study period but were not contingent on same-day use. Because anxiety reports reflected experiences over the prior 24 h, it is possible that the acute effects of flower use, which tend to be shorter in duration, had dissipated by the time participants completed their daily surveys. In contrast, this reporting window may have been better aligned with the longer-lasting effects of edibles, potentially capturing their extended influence on anxiety. Prior acute laboratory studies [13,38] support this distinction, showing that flower produces faster, shorter-lasting effects compared to the slower, prolonged effects of edibles. These findings underscore the importance of aligning assessment timing with the pharmacokinetics of different cannabis products when studying their daily effects on anxiety. Together, these patterns underscore how methods of use and cannabinoid composition jointly shape not just the quantity but also the temporal effects on anxiety, with CBD emerging as the most consistent driver of benefit.

### 4.4. Limitations and Future Directions

The present findings provide a novel contribution to the cannabis literature and leverage a naturalistic design comparing flower and edible products with a high degree of external validity. However, some limitations should be noted.

First, participants self-selected into flower or edibles groups. On one hand, this strengthens ecological validity by reflecting individuals’ naturalistic preferences and use patterns; on the other hand, it introduces potential bias, as pre-existing motivations or expectations about product type may have influenced both consumption behavior and perceived effects. To mitigate confounding associated with route self-selection, primary analyses were conducted separately for participants using flower and edible cannabis products. Nevertheless, residual confounding cannot be ruled out, as other factors (i.e., cannabis tolerance or timing of use relative to anxiety symptoms) may differ within routes and influence anxiety outcomes.

In addition, cannabis use may have varied as a function of daily anxiety, with participants choosing to use cannabis on more anxious days or abstain on less anxious days, which could influence comparisons between use and non-use days even within individuals. Similarly, missed daily surveys may have occurred more frequently on days with elevated anxiety or heavier cannabis use, although this cannot be directly evaluated in the present data. Relatedly, because this study did not include a placebo condition and relied on a naturalistic, observational design, the observed changes in anxiety over time and differences between use and non-use days should be interpreted as associational rather than causal.

Second, measurement and study design limit interpretation of the temporal specificity of the associations. Anxiety was assessed using once-daily self-report surveys reflecting the prior 24 h, which may be influenced by respondents’ affective state at the time of survey completion and may blur distinctions between immediate versus delayed anxiety responses following cannabis use. This limitation is particularly relevant given that participants were not blinded to the cannabis products they used, and expectations regarding THC- versus CBD-dominant products may have influenced self-reported anxiety despite covariate adjustment. Together, these factors limit inferences about short-term anxiety dynamics, especially for shorter-acting routes such as flower inhalation. Future studies incorporating ecological momentary assessment (EMA) could provide finer-grained, moment-to-moment data to better disentangle immediate versus extended effects, especially for shorter-acting methods like flower inhalation.

Third, cannabis use variables of dose and frequency were self-reported. Although this reflects real-world use patterns, estimates may be imprecise, and actual cannabinoid exposure may differ from reported values. In addition, product label accuracy for THC and CBD content may vary across products, introducing further measurement uncertainty. Laboratory testing of cannabinoid content and objective measures of exposure (i.e., blood samples) would strengthen future research.

Fourth, these analyses did not account for co-occurring interventions or behaviors that may influence anxiety, such as psychotherapy, medication use, alcohol or other substance use, exercise, or relaxation practices. These co-interventions may contribute to changes in anxiety over time and could not be disentangled from cannabis-related effects in the present analyses.

Finally, although data were collected in Colorado, a state with legal access to both medical and recreational cannabis, and our sample was relatively homogeneous (predominantly White and from higher socioeconomic backgrounds), which may limit the generalizability of the findings. Moreover, cannabis access, regulation, product availability, and prescribing practices vary substantially across states and countries, particularly for medical cannabis. Cannabis use may also pose risks for some individuals, including potential contraindications that could exacerbate psychiatric symptoms or contribute to cognitive or memory-related complaints. Future research should prioritize more diverse samples, include clinical populations, and explicitly examine how legal context, access pathways, and individual risk profiles shape mental health outcomes.

## 5. Conclusions

This study provides within-person, naturalistic findings that cannabis use is associated with short-term reductions in anxiety, with CBD products producing the most consistent benefits. The results suggest that, among individuals who already choose to use cannabis for anxiety, CBD-dominant products were associated with more consistent reductions in self-reported anxiety, although further placebo-controlled trials are needed. Public health messaging should highlight differences between THC and CBD products, as well as the greater variation regarding inhaled versus oral routes. Public health guidance must also account for substantial variability in access, regulation, and clinical oversight, as well as potential cognitive and mental health risks for some individuals. Broadly, this study highlights how real-world cannabis consumption patterns shape mental health outcomes and points toward more targeted approaches to therapeutic use.

## Figures and Tables

**Table 1:** Participant Demographics.

	Flower			Edible			*p*-Value (By Method of Use)
	CBD (*n* = 81)	THC + CBD (*n* = 73)	THC (*n* = 74)	CBD (*n* = 38)	THC + CBD (*n* = 39)	THC (*n* = 40)	
**Age**	30.0 ± 14.5	31.3 ± 16.2	30.7 ± 13.6	38.5 ± 14.5	36.3 ± 16.2	36.1 ± 13.6	<0.001
**Gender** (No. (%))							
Female	42 (51.9)	44 (60.3)	44 (59.5)	29 (76.3)	27 (69.2)	27 (67.5)	0.028
Male	36 (44.4)	29 (39.7)	29 (39.2)	9 (23.7)	11 (28.2)	11 (27.5)	
Non-binary	2 (2.5)	0 (0)	1 (1.3)	0 (0)	1 (2.6)	1 (2.5)	
Prefer not to answer	1 (1.2)	0 (0)	0 (0)	0 (0)	0 (0)	1 (2.5)	
**Education** (No. (%) Bachelor’s or higher)	46 (56.8)	31 (42.5)	39 (52.7)	27 (71.1)	29 (74.4)	29 (72.5)	<0.001
**Employment** (No. (%))							NS
Full- time employed	35 (43.2)	28 (38.4)	33 (44.6)	17 (44.7)	17 (43.6)	19 (47.5)	
Part-time employed	17 (21.0)	16 (21.9)	21 (28.4)	9 (23.7)	7 (17.9)	13 (32.5)	
Unemployed, disabled, retired, other	8 (9.9)	10 (13.7)	7 (9.4)	6 (15.8)	8 (20.5)	4 (10.0)	
Full- time student	20 (24.7)	17 (23.3)	13 (17.6)	6 (15.8)	7 (17.9)	3 (7.5)	
Homemaker	1 (1.2)	0 (0)	2 (2.7)	0 (0)	0 (0)	1 (2.5)	
**Ethnicity** (No. (%))							NS
American Indian/ Alaska Native	0 (0)	0 (0)	0 (0)	1 (2.6)	0 (0)	0 (0)	
Asian	4 (4.9)	3 (4.1)	4 (5.4)	0 (0)	1 (2.6)	1 (2.5)	
African American/Black	1 (1.2)	2 (2.7)	2 (2.7)	2 (5.3)	0 (0)	0 (0)	
White	63 (77.8)	56 (76.7)	47 (63.5)	31 (81.6)	36 (92.3)	36 (90.0)	
Hispanic/Latino	4 (4.9)	4 (5.5)	7 (9.4)	2 (5.3)	0 (0)	0 (0)	
More than one race/ethnicity	5 (6.1)	6 (8.2)	7 (9.4)	1 (2.6)	5 (12.8)	3 (7.5)	
Prefer not to answer	4 (4.9)	2 (2.7)	3 (4.1)	1 (2.6)	1 (2.6)	0 (0)	

Note: NS = non-significant *p*-value > 0.05. Age was reported as mean ± standard deviation, all other measures had number of participants (percentage within the group). All data reported in this table was collected during baseline appointment. Age ranged from 21 to 70 years old. The flower group was younger (*F* (1, 336) = 19.30, *p* < 0.001), more predominantly male (*X^2^* (2, *N* = 343) = 7.15, *p* = 0.028), and less educated (*F* (1, 339) = 21.30, *p* = 0.002) than the edibles group.

**Table 2:** Baseline Anxiety and Substance Use Descriptives.

	Flower			Edible			*p*-Value (By Method of Use)
	CBD (*n* = 81)	THC + CBD (*n* = 73)	THC (*n* = 74)	CBD (*n* = 38)	THC + CBD (*n* = 39)	THC (*n* = 40)	
Generalized anxiety disorder (GAD-7)	12.11 ± 4.16	11.28 ± 3.43	11.10 ± 4.25	11.08 ± 4.16	11.76 ± 4.12	12.18 ± 4.37	NS
Cannabis anxiety expectancy	1.01 ± 0.75	1.12 ± 0.82	1.08 ± 0.66	0.92 ± 0.43	1.10 ± 0.64	1.00 ± 0.60	NS
Cannabis use disorder (CUD) symptoms	2.06 ± 2.28	2.06 ± 2.50	2.08 ± 2.14	0.92 ± 1.85	0.61 ± 0.95	0.78 ± 1.34	<0.001
**Days of substance use over 14 days prior to baseline**							
Cannabis use	6.47 ± 5.19	6.18 ± 5.09	6.45 ± 5.10	3.76 ± 4.28	4.03 ± 5.18	2.68 ± 3.85	<0.001
Flower cannabis use	4.37 ± 4.99	5.33 ± 5.22	4.72 ± 4.73	0.89 ± 2.47	1.00 ± 3.21	0.60 ± 1.84	<0.001
Edible cannabis use	0.93 ± 2.23	0.76 ± 1.64	0.97 ± 2.11	2.16 ± 3.18	2.30 ± 3.76	0.75 ± 1.35	0.002
Alcohol use	2.91 ± 2.86	3.56 ± 3.57	2.96 ± 2.77	3.11 ± 3.11	3.73 ± 3.64	3.57 ± 3.58	NS

Note: NS = non-significant *p*-value > 0.05. Results are reported as mean ± standard deviation. All data reported in this table was collected during baseline appointment. GAD-7 scores ranged from 4 to 21. Cannabis expectancies ranged from 0 to 4. Cannabis use disorder (CUD) symptoms were measured with the Marijuana Dependence Scale (MDS) and scores ranged from 0 to 11. The flower group had more symptoms than the edibles group *F* (1, 334) = 29.75, *p* < 0.001. Days of substance use was measured using the Timeline Follow Back (TLFB). The flower group used more cannabis products (*F* (1, 334) = 26.32, *p* < 0.001), more flower-based products (*F* (1, 334) = 64.15, *p* < 0.001), and fewer edible products (*F* (1, 334) = 9.46, *p* = 0.002) than the edibles group. No significant difference was observed within the flower and edibles groups.

**Table 3:** Cannabis Study Product Use.

	Flower			Edible			*p*-Value
	CBD (*n* = 81)	THC + CBD (*n* = 73)	THC (*n* = 74)	CBD (*n* = 38)	THC + CBD (*n* = 39)	THC (*n* = 40)	
Days of study product use	14.46 ± 7.58	15.99 ± 7.51	16.26 ± 8.26	14.71 ± 7.35	13.64 ± 7.13	12.95 ± 7.82	NS
Total number of times participants used their product over the study	26.69 ± 23.30	30.14 ± 23.95	35.95 ± 35.80	18.82 ± 12.02	18.31 ± 18.54	17.40 ± 15.09	<0.001
Dose (g)	0.23 ± 0.41	0.21 ± 0.35	0.20 ± 0.78	N/A	N/A	N/A	NS
THC Dose (mg)	N/A	N/A	N/A	1.85 ± 3.19	5.58 ± 4.22	8.28 ± 18.54	<0.001
CBD Dose (mg)	N/A	N/A	N/A	25.25 ± 24.81	3.35 ± 2.43	5.61 ± 4.27	<0.001

Note: NS = non-significant *p*-value > 0.05. Results are reported as mean ± standard deviation. All data reported in this table was based on daily data. Dose data was self-reported by participants. For days of study product use and the total number of times participants used their product over the study, the *p*-value is based on method of use. The flower group used their products more times than the edibles group. For dose data, the *p*-value is based on cannabinoid type.

## Data Availability

The datasets used and/or analyzed during the current study are available from the corresponding author on reasonable request.

## Abbreviations

The following abbreviations are used in this manuscript:

THC	9-tetrahydrocannabinol
CBD	Cannabidiol
CUD	Cannabis Use Disorder
GAD-7	Generalized Anxiety Disorder Scale
